# Differential Mechanical Response of Mesenchymal Stem Cells and Fibroblasts to Tumor-Secreted Soluble Factors

**DOI:** 10.1371/journal.pone.0033248

**Published:** 2012-03-16

**Authors:** Daniel J. McGrail, Deepraj Ghosh, Nhat D. Quach, Michelle R. Dawson

**Affiliations:** 1 School of Chemical & Biomolecular Engineering, Georgia Institute of Technology, Atlanta, Georgia, United States of America; 2 School of Chemistry and Biochemistry, Georgia Institute of Technology, Atlanta, Georgia, United States of America; 3 The Petit Institute for Bioengineering and Bioscience, Georgia Institute of Technology, Atlanta, Georgia, United States of America; Dalhousie University, Canada

## Abstract

The progression of neoplastic malignancies is a complex process resulting not only from the accumulation of mutations within tumor cells, but also modulation of the tumor microenvironment. Recent advances have shown that the recruitment and subsequent heterotypic interactions of stromal cells—including fibroblasts and bone marrow-derived mesenchymal stem cells (MSCs)—are crucial for carcinogenesis. Though extensive work has been done analyzing the signals that recruit these cells, the governing mechanical properties have not been fully investigated. Here, we report that despite their initial similarities, MSCs respond not only faster but also more dramatically to pro-migratory tumor-secreted soluble factors. Utilizing multiple particle tracking microrheology to probe the cytoskeletal mechanical properties, we show that MSCs stiffen completely within one hour, three times faster than fibroblasts. In addition, unlike fibroblasts, MSCs exposed to tumor-secreted soluble factors display a functionally different phenotype characterized by morphological elongation, decreased actin stress fiber density, and decreased adhesion. Quantitative real-time PCR indicates these phenomena occur based on differential expression of small GTPases RhoA and Cdc42, but not Rac1. These findings demonstrate a fundamental difference in the recruitment of fibroblasts and MSCs.

## Introduction

The microenvironment in a solid tumor develops under the constant influence of inflammatory mediators [1]. These molecules, which include a milieu of cytokines, chemokines, and growth factors, are important targets for recruitment of a variety of cells such as leukocytes, macrophages, monocytes, fibroblasts and mesenchymal stem cells (MSCs) [2]. Previous literature on MSCs and fibroblasts suggests functional similarity as indicated both by global gene expression [3] as well as immunosuppression in allogeneic transplantation [4]. In the tumor stroma both can also form activated cancer-associated fibroblasts (CAFs) or myofibroblasts [2], [5], though MSCs can additionally differentiate into pericyte progenitor cells (PPCs) [6]. Increased numbers of myofibroblasts in the wound bed and in other sites of chronic inflammation have also been associated with MSC progenitors [2]. Notably, both cell types also aid in tumor growth and metastasis via autocrine and paracrine signaling [7], [8]. In light of this, recent studies have begun to investigate these cells not only as alternative targets for anti-cancer therapy [9], but also for use as targeted gene-delivery vehicles [10].

For the latter approach, MSCs have shown greater promise than fibroblasts [11]. This may be in part because fibroblasts are recruited locally to form activated CAFs [12], whereas MSCs derived from the bone marrow must natively home through circulation to distal tumor sites. Consequentially, the therapeutic use of systemically infused MSCs to tumors has been investigated in breast [13], colon [14], ovarian carcinomas [15], gliomas [16], and Kaposi's sarcomas [17]. Despite this potential, extended *ex vivo* culture reduces homing capacity of MSCs [18] and the majority of systemically infused MSCs become trapped in the lungs [5], [19], [20]. To overcome this, breast cancer cell-conditioned media [7], hypoxic preconditioning [21], [22], and treatment with individual chemokines or growth factors [23], [24] have all been investigated to increase MSC mobility; however, the effects of inflammatory mediators on the microscopic mechanical properties of MSCs have not been fully elucidated.

The mechanical properties within the cell are largely regulated by the actin cytoskeleton, a complex network of interconnected actin filaments and regulating proteins [25], [26]. Dynamic changes in the organization of the cytoskeleton transform cell shape and generate mechanical forces required for numerous cellular processes, including adhesion, migration, division, molecular transport, and differentiation [27]. Cytoskeletal effector proteins coordinate these changes by polymerization, depolymerization, cross-linking, and bundling of actin filaments into actin stress fibers, lamellipod extensions, and actin networks. Parallel bundles of actin filaments provide tensile strength and strong contractile activity, whereas cross-linked bundles of actin filaments increase intracellular elasticity [27]. Chemical and physical stimuli have been shown to alter cell shape and cytoskeletal organization by activating cytsokeletal mediators, including RhoA, Rac1, and Cdc42 [28], [29], [30]. Recent studies have highlighted the importance of mechanical [31] and chemical [32] cues on MSC fate: in these studies, soluble factors [32], cell shape [32], and extracellular matrix rigidity [31] regulated the lineage commitment of MSCs through RhoA signaling pathways.

This study sought to understand the underlying mechanical differences in MSCs and fibroblasts as they migrate toward tumors. In order to best simulate the signals migrating cells would receive *in vivo*, we utilized 4T1 breast tumor cell conditioned media (TCM) to stimulate the cells *in vitro*. We found that after treatment with TCM, MSCs underwent an exaggerated response as compared to fibroblasts, and that this response could potentially be explained by altered gene expression of Rho GTPases Cdc42 and RhoA. We further demonstrate that even one hour incubation with TCM increases cell motility, indicating a novel ‘mechanical priming’ achieved by short-term TCM preconditioning.

## Results

### Characterization of Bone Marrow Derived MSCs

**Figure 1:**
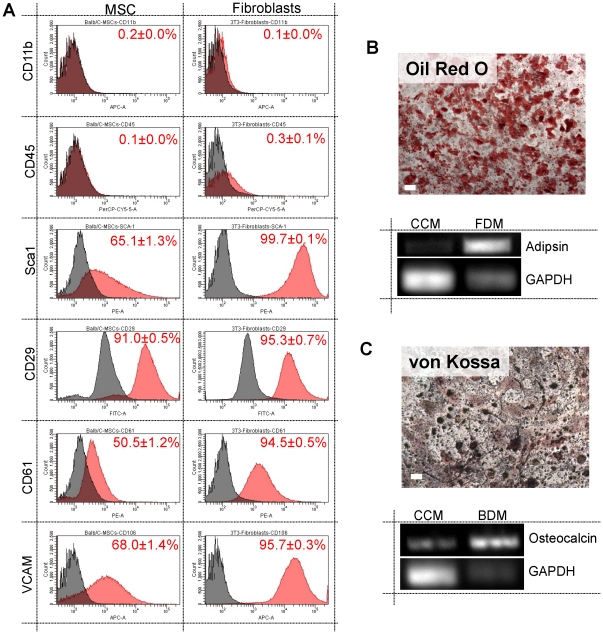
Characterization of bone marrow isolated MSCs and fibroblasts. Phenotypic analysis was performed by flow cytometry was performed on adherent bone marrow cells and Swiss 3T3 fibroblasts with positive populations in red given with S.E.M.(A). Purified MSCs differentiated into adipocytes (B) and osteoblasts (C) within 3 weeks in lineage-specific differentiation media as shown both my staining and RT-PCR (scale bar = 100 µm).

To verify the phenotype of MSCs, cells were assayed for both cell surface marker expression and differentiation capacity. After purification by adherence to plastic, MSCs were negative for myeloid and hematopoietic stem cell markers CD11b and CD45 and positive for Sca1, CD29, CD61, and VCAM1 (Fig. 1A, Left). Fibroblast expression is provided for comparison (Fig. 1A, Right). Osteogenesis and adipogenesis were induced in passage 4 MSCs using standard protocols [33]. After 4 week incubation in either adipogenic media or osteogenic media, MSCs differentiated into adipocytes (Fig. 1B) and osteoblasts (Fig. 1C) as verified by histological staining and reverse transcriptase PCR [34].

### Morphological Changes after Incubation with Tumor Cell-Secreted Soluble Factors

**Figure 2:**
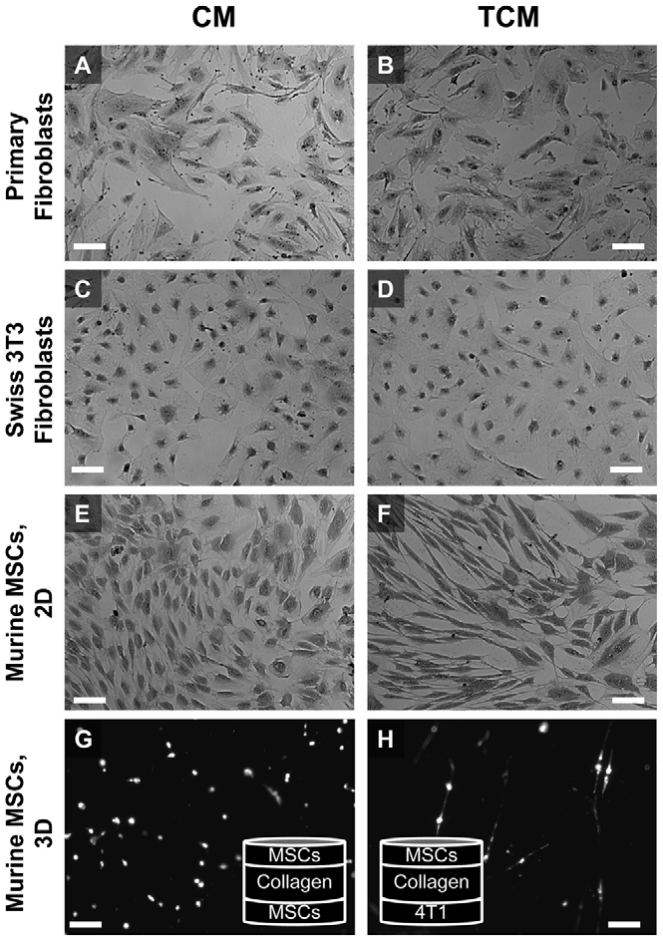
Tumor-secreted soluble factors alter MSC morphology. Brightfield images of murine MSCs (isolated from Balb/C mouse bone marrow; A–B), Swiss 3T3 fibroblasts (C–D), and primary fibroblasts (isolated from Balb/C mouse kidney; E–F) incubated for 24 hours in control media (CM, top) or tumor cell-conditioned media (TCM, bottom), then fixed in methanol, and stained with crystal violet. MSCs elongated dramatically in response to TCM (A–B); whereas, immortalized (C–D) and primary (E–F) fibroblasts did not respond to TCM treatment. A ‘sandwich’ co-culture model was used to study the migratory behavior of CM-DiL-labeled MSCs through a layer of polymerized collagen toward a monolayer of MSCs (G) or 4T1 tumor cells (H). Interestingly, MSCs elongated toward the tumor cells (H) but not toward the MSCs. (scale bar = 100 µm)

To investigate the response of MSCs to tumor cell-conditioned media (TCM), cells were incubated in serum-free TCM or control media (CM) for 24 hours. MSCs underwent a morphological change from a cobblestone appearance to an elongated phenotype (Fig. 2A–B). In comparison, Swiss 3T3 fibroblasts (Fig. 2C–D) and primary isolated kidney fibroblasts (Fig. 2E–F) had similar initial morphologies, but did not undergo a morphological change upon treatment with TCM. A 3D sandwich culture model was used to determine if tumor cells could stimulate elongation through a viscoelastic collagen gel. Moreover, MSCs elongated through the collagen gel toward a monolayer of 4T1 tumor cells (Fig. 2H) but did not elongate towards a monolayer of MSCs (Fig. 2G).

### Cytoskeletal Changes after TCM Treatment

**Figure 3:**
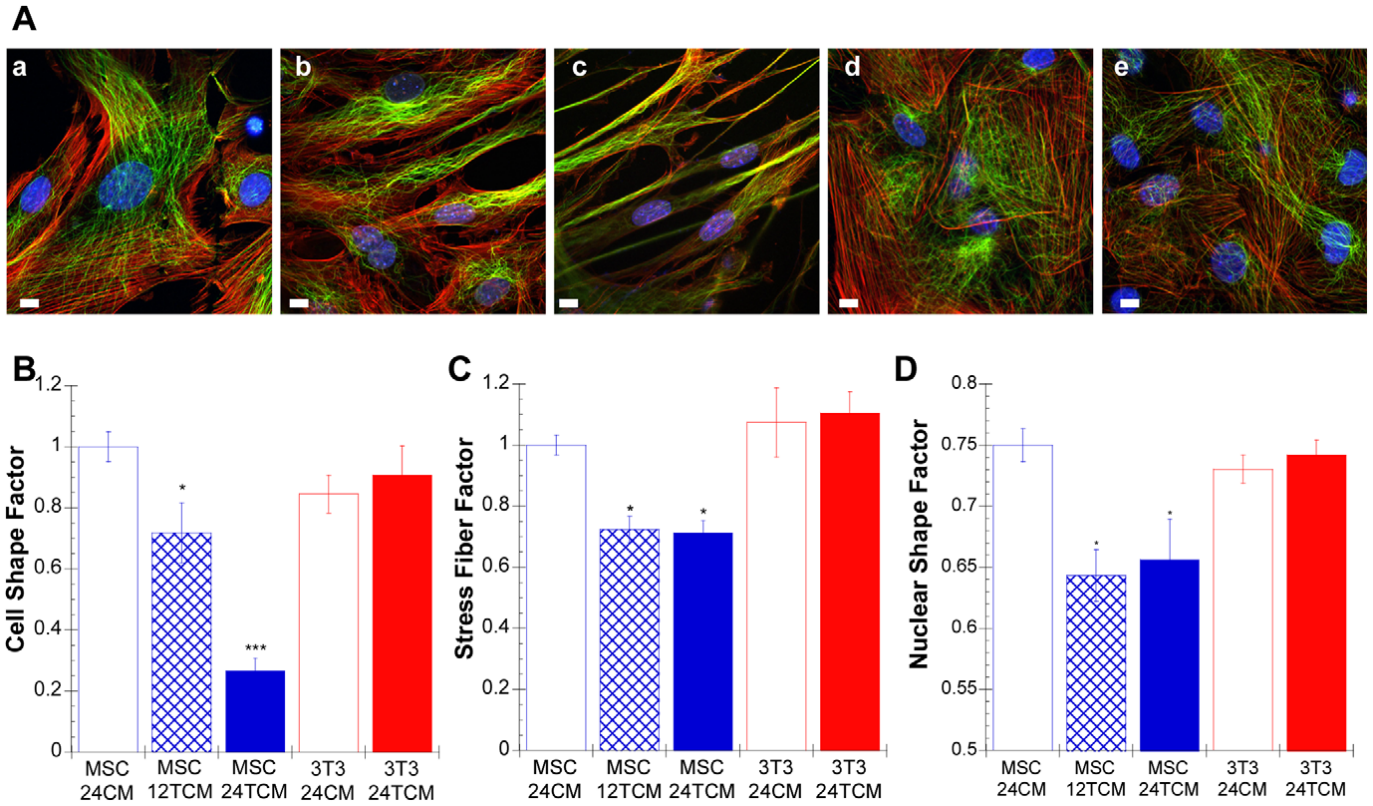
MSCs reorganize their cytoskeleton in response to tumor-secreted soluble factors. (A) Confocal micrographs of CM and TCM-treated MSCs (a–c) and 3T3 fibroblasts (d–e) stained with Phalloidin (F-actin, red), anti-α-tubulin (microtubules, green), and DAPI (nucleus, blue). The shape and cytoskeletal organization of CM-treated MSCs (a) and CM- (d) and TCM- (e) treated Swiss 3T3 fibroblasts were similar (24 hours after CM or TCM addition); whereas, TCM-treated MSCs were elongated with extended cytoskeletal filaments (b–c). MSC elongation increased between 12- (b) and 24- (c) hours, indicating that cytoskeletal changes may be progressive. Cytoskeletal parameters (B–D) were determined by analysis of confocal images with a custom MATLAB routine. The cell (B) and nuclear (D) shape factors were used to characterize the circularity of an elliptical outline of the cell or nucleus, respectively, with a shape factor of 1 indicating a perfect circle. The stress fiber factor (C) was used to characterize the density of actin stress fibers per cell area. Cytoskeletal changes observed in TCM-treated MSCs (b–c) were confirmed using the cytoskeletal parameters (B–D), which indicated dramatic reductions in cell and nuclear shape factors and stress fiber densities. (scale bars = 10 µm)

We next examined the changes in cytoskeletal arrangement necessary to produce the elongated phenotype. To accomplish this, CM and TCM treated cells were fixed and stained for filamentous actin, microtubules, and nuclei (Fig. 3A). Cytoskeletal parameters were quantified from maximum intensity projections of confocal image stacks (3 images per stack) using a custom-written MATLAB algorithm. First, the cell elongation factor was quantified using a ratio of the minor to major cell axis (Fig. 3B). For control MSCs and 3T3 fibroblasts, the cell shape factors were near unity, indicating similarity in their native cell shape. In the presence of TCM, MSCs elongated by ∼30% after 12 hours (p<0.05) and ∼75% after 24 hours (p<0.001); whereas, the elongation factor of 3T3 fibroblasts remained near unity. We then quantified the stress fiber factor, which is the density of stress fibers normalized to cell area (Fig. 3C). The stress fiber factor of TCM-treated 3T3 fibroblasts remained constant; whereas, the stress fiber factor of MSCs was reduced more than 25% within 12 hours. Though this could in part be due to control cells being flatter than TCM treated cells, the use of maximum intensity projections minimizes this possibility, suggesting the changes are in fact due to decreases in actin stress fiber density. Upon stimulation with TCM, the nuclei of MSCs elongated dramatically (Fig. 3A). A nuclear shape factor was used to characterize nuclear elongation (Fig. 3D), with a value of one indicating the nucleus is perfectly round. Initially, MSCs and 3T3 fibroblasts had nuclear shape factors of approximately 0.75; however, upon incubation with TCM for 12 hours, MSC nuclei were elongated by approximately 13% (Fig. 3D). Nuclear elongation was never seen in 3T3 fibroblasts and no further elongation was observed in MSCs from 12–24 hours (Fig. 3D).

### TCM Alters the Distribution and Strength of MSC Focal Adhesions

**Figure 4:**
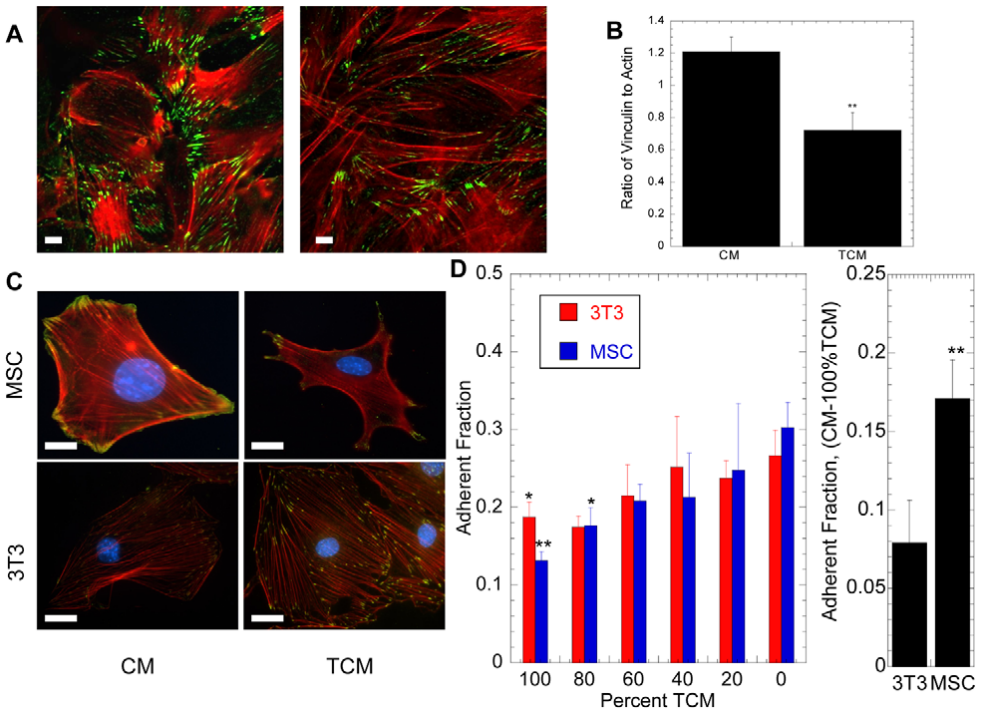
Changes in the distribution and strength of focal adhesions. Confocal micrographs (A) of 24-hour CM- (left) and TCM- (right) treated MSCs stained with Phalloidin (F-actin, red), and anti-vinculin (green). The vinculin to actin ratios quantified from confocal images (B) demonstrated a reduced concentration of focal adhesion proteins in TCM-treated MSCs. Epifluorescent microscopy was used to further investigate the effect of TCM on focal adhesion distribution in MSCs and 3T3 fibroblasts (C). CM-treated MSCs displayed a brush-like pattern of focal adhesions; whereas, focal adhesion on TCM-treated cells appeared as points at the end of cytoskeletal extensions. TCM-treatment had no effect on the pattern of focal adhesions on 3T3 cells. A centrifuge-based adhesion assay was used to determine the effects of TCM-treatment on the adhesion of MSCs and 3T3 fibroblasts (D). TCM treatment resulted in a reduced fraction of adherent cells in a dose-dependent manner for both cell type (left), but significantly more for MSCs (right). (scale bars = 10 µm)

The cytoskeleton is linked to the extracellular environment via focal adhesion complexes, which are important regulators of cell motility. To determine the effects of TCM on cell adhesion, control and TCM-treated MSCs and 3T3 fibroblasts (treated for 24 hours) were fixed and stained with FITC anti-vinculin, a focal adhesion marker, and Rhodamine-Phalloidin, which stains filamentous actin (Fig. 4A,C). TCM treatment dramatically affected the overall number and morphology of focal adhesions on MSCs (Fig. 4A–C); however, it had no apparent effect on 3T3 fibroblast focal adhesions (Fig. 4C). For MSCs, the ratio of vinculin to actin was quantified from confocal images using a MATLAB routine (Fig. 4A,B). Twenty-four hours after TCM addition, the vinculin to actin ratio was reduced by 50% (Fig. 4B), and focal adhesions no longer displayed their characteristic brush-like pattern (Fig. 4B–C) but were instead localized at the tips of elongated cells (Fig. 4A,C).

A centrifuge-based adhesion assay was used to determine if changes in vinculin expression were correlated with changes in cell adhesion (Fig. 4D). MSCs incubated for 24 hours with 80% or 100% TCM were significantly less adhesive, with a 36% or 54% reduction in the adherent cell fraction, respectively. Incubation with 100% TCM also reduced 3T3 fibroblast adhesion by 24%. Changes in the adherent fraction were only significant at high TCM concentration (≥80%), indicating that the adhesive response to tumor-secreted soluble factors would be most important near the tumor.

### Cytoskeletal Stiffening in Response to TCM

**Figure 5:**
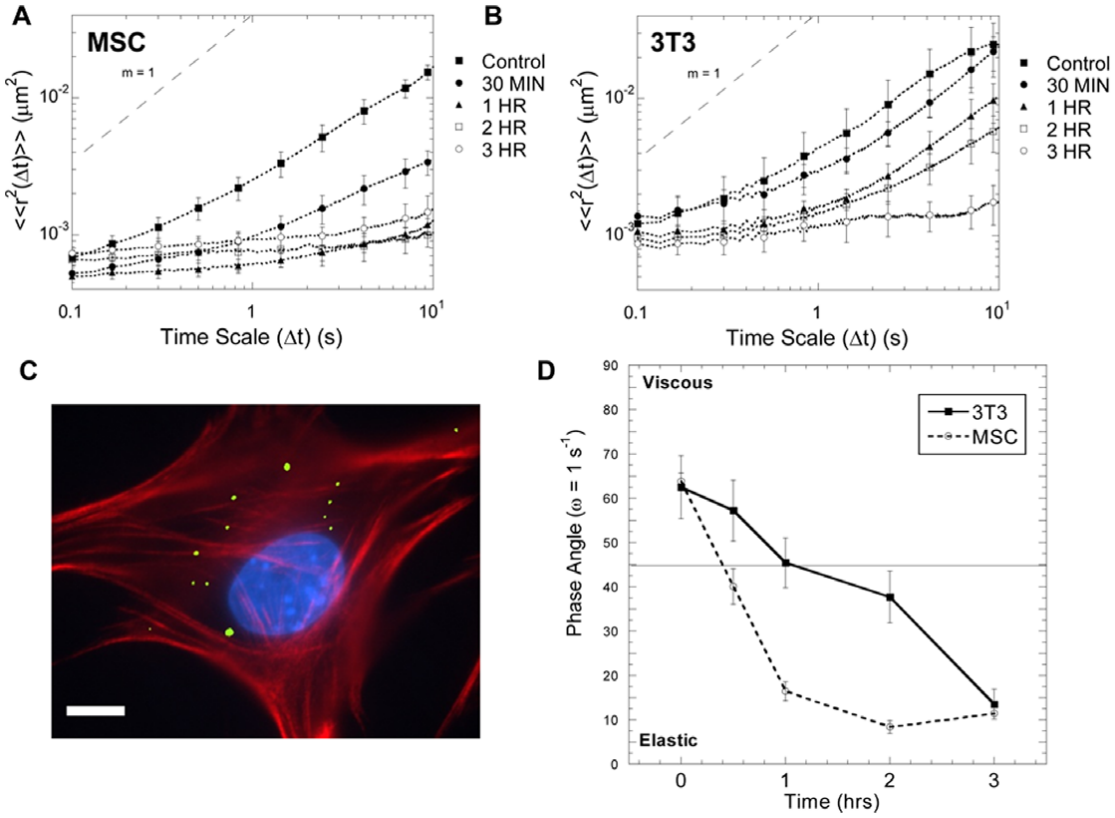
Multiple particle tracking microrheology. The ensemble averaged mean squared displacements (<<r^2^(Δt)>>) of 100 nm particles embedded in the cytoplasm of TCM-treated MSCs (A) and 3T3 fibroblasts (B) were evaluated from 0–3 hours. For both cell lines, treatment with TCM reduced the rate of cytoplasmic particle transport in a time-dependent manner. Fluorescent image of 100 nm particles (green) in the cytoplasm of a MSC, which was fixed and stained with phalloidin (red) and DAPI (blue) (C). The phase angle, δ = arctan (G″(ω))/G′(ω)), was used to characterize the viscoelastic nature of the cytoplasm over the course of the experiment (D). The viscoelastic nature of MSCs and 3T3 fibroblasts were similar initially and 3 hours after TCM-treatment; however, MSCs responded much more rapidly to TCM with a 4-fold reduction in δ within 60 minutes. (scale bar = 10 µm)

**Figure 6:**
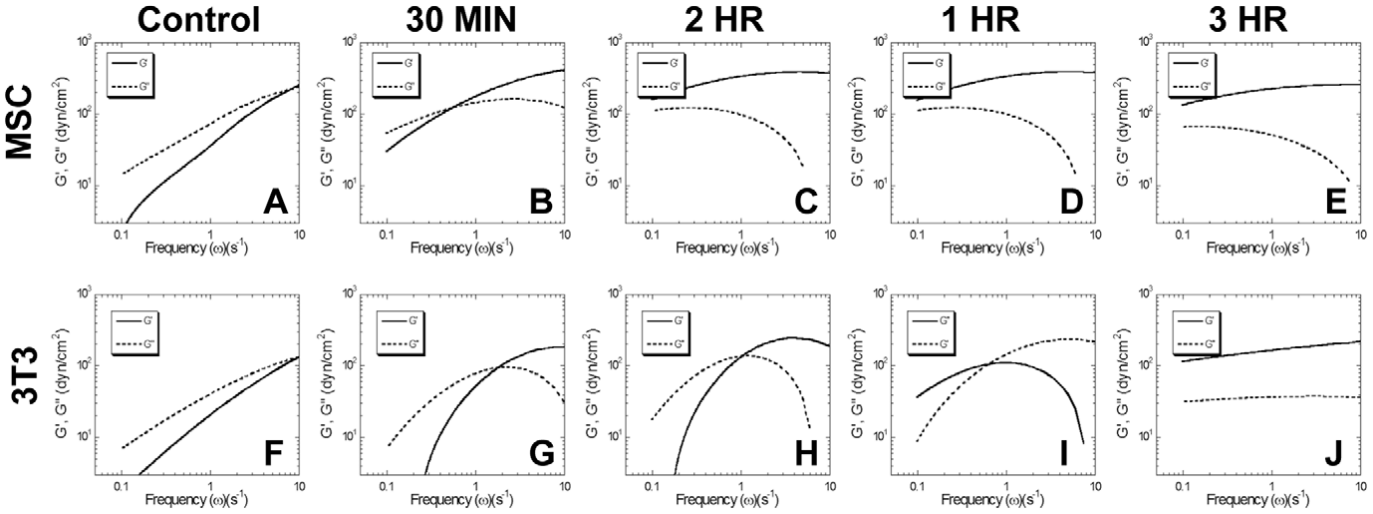
TCM alters cytoplasmic rheology. The time-dependent ensemble averaged MSDs of 100 nm particles embedded in the cytoplasm of MSCs and 3T3 fibroblasts were converted to frequency-dependent elastic (G′, solid lines) and visous (G″, dashed lines) moduli using a custom algorithm written for Matlab software. The ensemble-averaged frequency-dependent viscoelasticities of MSCs (A–E, left) and 3T3 fibroblasts (F–J, right) prior to (A,F) and 30 minutes (B,G), 1 hour (C,H), 2 hours (D,I), or 3 hours (E,J) after treatment with TCM. The cytoplasm of MSCs became predominantly elastic within 60 minutes; whereas, 3T3 fibroblasts required 3 hours to undergo a similar change.

MPT was used to characterize the immediate effects of TCM (30 minutes to 3 hours) on the intracellular mechanical properties of MSCs (Fig. 5A, Fig. 6A–E) and 3T3 fibroblasts (Fig. 5B, Fig. 6F–J). Corresponding morphological changes were not seen in immunostained fixed MSCs until 12–24 hours after TCM treatment and were never seen in 3T3 fibroblasts (Fig. 3–4). For MPT studies, 100 nm probe particles, with diameters more than 100-fold smaller than the cells, were injected into the cytoplasm (Fig. 5C), and their mobility, characterized by the <<Δr^2^(Δt)>> (Fig. 5A–B), was used to determine intracellular rheology.

Initially, the amplitude and logarithmic slope of the <<Δr^2^(Δt)>> for MSCs and 3T3 fibroblasts were comparable, indicating similarity in their microscopic mechanical properties (Fig. 6A,F). Within 30 minutes, TCM treatment reduced the <<Δr^2^(Δt)>> for MSCs up to 20-fold; however, similar changes were not seen in 3T3 fibroblasts until 2–3 hours after TCM treatment. The ratio of viscous to elastic character, or phase angle (δ), of control cells was 55°<δ<70°, indicating that initially the cytoplasm behaved more like a viscous liquid than an elastic solid. One hour after TCM treatment, the cytoplasm of MSCs had dramatically stiffened with 10°<δ<15°. This effect was not seen in 3T3 fibroblasts until 3 hours after TCM treatment. MPTM was also used to quantify the rheological properties of the cytoplasm, which is highly viscoelastic in part to its crowded nature. The frequency-dependent viscoelasticity of MSCs changed dramatically after TCM treatment (Fig. 6A–E). In fact, the cytoplasm became highly elastic with G′∼400 dyn/cm^2^ (Fig. 6B–E).

### TCM Rapidly Increases In Vitro Cell Migration

**Figure 7:**
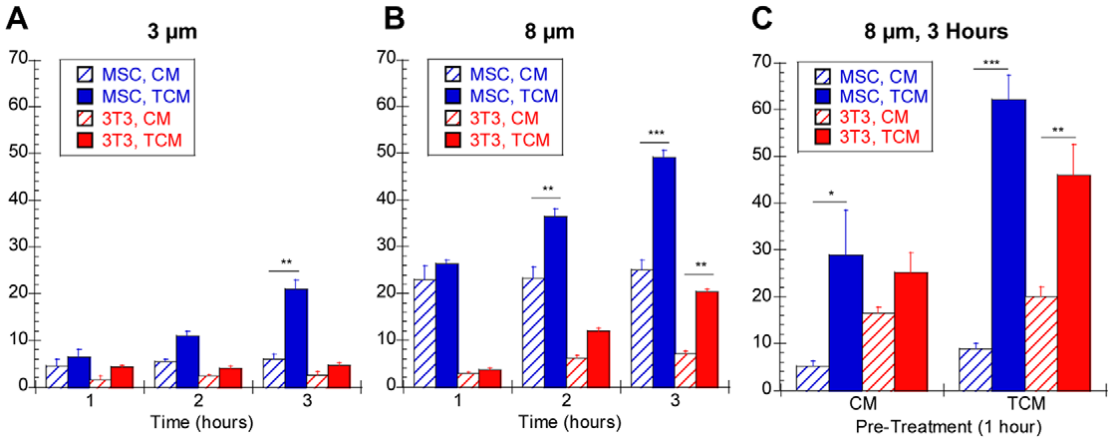
Effect of TCM on Cell Migration. Tranwell assays were used to measure the migration of MSCs and 3T3 fibroblasts through 3 µm- (A) and 8 µm- (B) pore transwell inserts toward CM or TCM. The average number of cells per image (n = 9), collected with a 10×-objective, was reported. TCM significantly increased MSC migration, compared to CM, through 3 µm pores within 3 hours and 8 µm pores within 2 hours; however, fibroblast migration was only increased through 8 µm pores within 3 hours. MSCs and 3T3 fibroblasts were then treated with CM or TCM for 1 hour and allowed to migrate through 8 µm-pore transwell inserts toward CM or TCM for 3 hours (C). Pre-treatment with TCM resulted in synergistic effects on chemotactic migration for both cell types.

**Figure 8:**
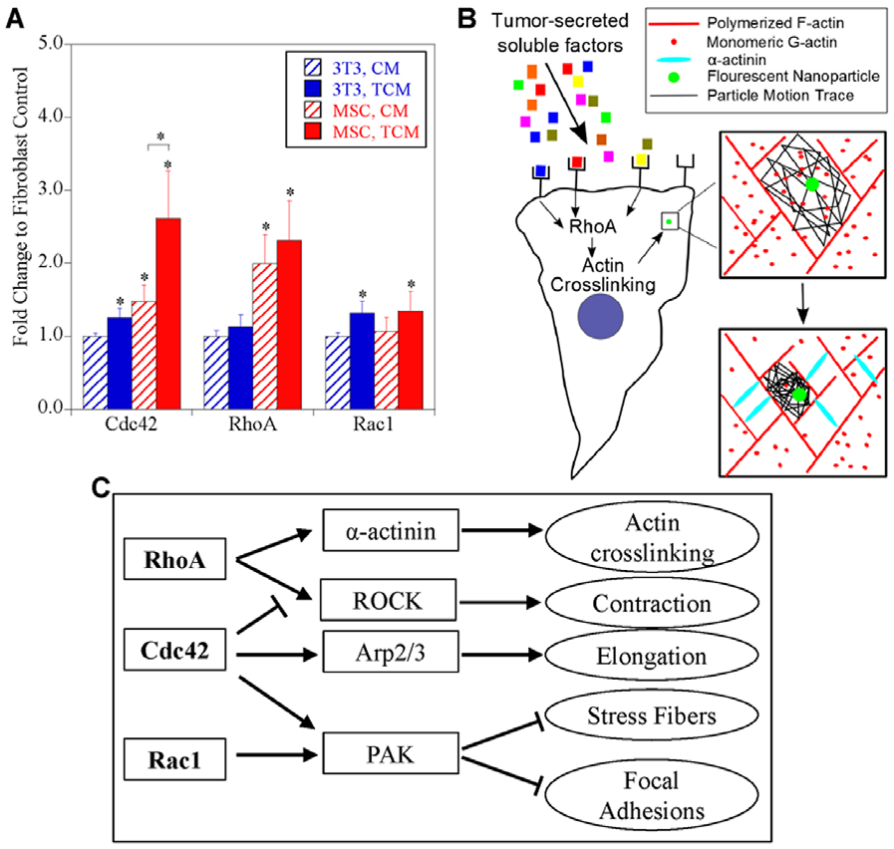
Alterations in Gene Expression. Changes in expression of small Rho GTPases RhoA, Rac1, and Cdc42 before and after prolonged treatment with TCM (A). A simplified diagram of short-term response of cells to treatment with TCM by actin polymerization and crosslinking (B). After 24 hour exposure to TCM, alterations in morphology and adhesion can be explained by differential gene expression in MSCs and fibroblasts (C).

Previous studies documented increased MSC migration toward chemotactic factors after 18–24 hours [23], [24], [35]. Our multiple particle tracking studies revealed dramatic changes in intracellular mechanics within 1 hour. To determine if these mechanical changes were correlated with increased mobility, the migration of control and treated cells toward CM or TCM was measured hourly for 3 hours using transwell inserts with 3 µm (Fig. 7A) or 8 µm (Fig. 7B) pores. Without chemotactic factors present, few MSCs and 3T3 fibroblasts migrated through 3 µm pores; however, when TCM was added, there was a significant increase in MSC migration within 3 hours (p<0.01, Fig. 7A). MSC migration through 8 µm pores was almost always significantly greater than 3T3 fibroblast migration (Fig. 7B–C)). MSCs also responded more rapidly to TCM, with increased MSC migration within 2 hours (p<0.001) and increased 3T3 fibroblast migration within 3 hours (p<0.01). To determine if mechanical changes exhibited by MSCs in the first hour were indicative of a more migratory phenotype, we pre-treated both cell types for one hour with TCM before seeding in 8 µm transwell inserts. Pre-treatment with TCM increased MSC and 3T3 fibroblast migration toward CM and TCM, though more significantly toward TCM (Fig. 7C). Statistical analysis also revealed synergistic effects of TCM pre-treatment and migration toward TCM for both cell types, suggesting that pre-treatment not only increases motility but also causes a preferential migration towards chemotactic gradients.

### Changes in Gene Expression Associated with Altered Mechanical Response

To understand genotypic changes resulting in altered mechanical responses, we performed qRT-PCR to investigate the expression of the Rho GTPases RhoA, Rac1, and Cdc42 that modulate the actin cytoskeleton (Fig. 8A). We found that after TCM treatment, both cell types express significantly more Rac1 (p<.05) as compared to their respective controls. Furthermore, ANOVA revealed that MSCs expressed significantly more RhoA overall (p<.0001). Moreover, it showed a significant interaction effect between cell type and TCM treatment for Cdc42 (p<.005), suggesting that this molecule is largely responsible for the altered mechanical response.

## Discussion

In this study, TCM isolated from 4T1 metastatic breast cancer cells was used to mimic the chemotactic factors released by tumors. Previous studies have demonstrated that TCM is a potent pro-migratory cocktail of growth factors and chemokines [35], [36] (for a detailed list of acronyms see Table S1). Literature on 4T1 tumor cell conditioned media shows that it contains soluble growth factors including platelet derived growth factor (PDGF), transforming growth factor (TGF-β1), and vascular endothelial growth factor (VEGF) [37], as well as cytokines, chemokines, acute phase proteins and proteases. In two separate studies [38], [39], researchers examined the gene expression profile of 4T1 tumor cells in comparison to less metastatic cell lines, finding a variety of up-regulated pro-migratory soluble factors including (a) growth factors angiopoietin 2, VEGF-C, insulin-like growth factor 2, (b) chemokines CCL5, CCL7, CXCL1, CXCL16, CSF2, CSF3, (c) interleukins 1α and 23, and (d) matrix metalloproteases 13,3,9. Yet this list is not comprehensive, as the complexity of TCM continues to yield novel pro-migratory soluble factors [36] in addition to unique miRNA secretomes [40].

Some of these growth factors, such as PDGF, TGF- β1, and VEGF, have been shown to directly induce mechanical and migratory changes in MSCs and fibroblasts [23], . However, the effects of other soluble factors present in TCM are often much more difficult to determine. Menon et al. found that MSC exposure to tumor cell conditioned media up-regulates mRNA levels of 104 genes, including genes for stromal cell-derived factor 1 (SDF-1/CXCL12), monocyte chemotactic protein 1 (MCP-1/CCL2), and growth-regulated protein β (Gro-β/CXCL2), which are all potent chemokines that act in an autocrine fashion to further stimulate MSC migration [35]. In the same study, Menon et al. also found that TCM altered the organization of cytoskeletal actin, resulting in increased polarity and directional migration [35]. In another study, soluble factors (identified as extracellular matrix peptides) in LLC1 tumor cell conditioned media induced bone marrow derived cell secretion of pro-inflammatory cytokines, including IL-1β, IL-6, and tumor necrosis factor α (TNF-α), whereas serum-free media and 3T3 conditioned media had no effect on cytokine production [44]. In contrast, tumor cells exchange paracrine signals with normal fibroblasts, which have been shown to promote matrix remodeling and tumor cell invasiveness [41]. One way this occurs is through tumor cell secretion of soluble factors, such as IL-1, FGF-2, PDGF, and/or TGF- β, which induce hepatocyte growth factor (HGF) secretion from fibroblasts. HGF then binds with its cognate receptor, c-MET, which is expressed by normal and cancer stem cells [45]. The HGF/c-MET signaling pathway triggers cell growth and angiogenesis, which are important mechanisms of cancer development, normal growth, and wound healing [41].

We hypothesize that several of the growth factors and chemokines in TCM, such as VEGF, PDGF, TGF- β1, directly stimulate changes in MSCs and fibroblasts; however, other factors in TCM, such as IL-1β, colony-stimulating factor (CSF), and TNF-α are more potent mediators of changes in MSCs due to autocrine signaling pathways including SDF-1, MCP-1, and Gro-β, which they activate rapidly. Interestingly, a more detailed analysis of MSC migration to individual growth factors and chemokines found that TNF-α stimulation was necessary for MSC migration towards all chemokines tested, but had little effect on the migration of MSCs toward growth factors [23]. Due to the complicated nature of the autocrine and paracrine signaling pathways involved in the recruitment of cells to tumors, we focused our study on characterizing the effects of a cocktail of these factors in TCM on MSC and fibroblast behavior.

The molecular response of MSCs to various soluble factors has been well characterized, but the mechanical properties that govern their migration are yet to be fully investigated. MPTM was used to analyze the real-time changes in live-cell mechanics. Previous MPT studies have demonstrated not only that induced pluripotent stem cells differ with respect to intracellular rheology from their parental fibroblasts line [46], but also that fibroblasts stiffen both during migration and in response to activation of RhoA, Cdc42, or Rac1, with the largest response to RhoA [25], [42]. This technique revealed cytoskeletal stiffening after TCM treatment larger than previously documented, completely changing the intracellular mechanical phenotype, three-fold faster in MSCs (Fig. 5, 6).

Analysis of expression of RhoA in untreated MSCs and fibroblasts revealed two-fold higher expression in MSCs (Fig. 8A), which could account for more rapid cytoskeletal stiffening of MSCs. RhoA can act by Rho Kinase (ROCK) to induce stress fiber formation and focal adhesions or to crosslink actin filaments via the downstream effector α-actinin [47]. In MSCs, inhibition of ROCK has been shown to increase cell motility, an effect which is increased in presence of the RhoA activator lysophosphatidic acid (LPA) [48]. In fibroblasts, equivalent stiffening to that induced by TCM was found by inhibition of ROCK before RhoA activation [25]. Moreover, this increase in elastic character is in good agreement with *in vitro* experiments analyzing actin polymerization and crosslinking dynamics [49]. A simplified schematic of this process can be seen in Figure 8B.

Transwell migration assays further illustrated how crucial this stiffening was for chemotaxis, as significant migration to TCM over control media was not achieved until the time point after cells had transitioned to a primarily elastic phenotype (Fig. 7B). Additionally, *in vitro* migration assays show that even a one hour exposure to TCM increases MSC migration. Since soluble factors mediate cell migration by interacting with cell surface receptors that require much longer synthesis and transport times [50], these results suggest a novel ‘mechanical priming’ achieved by short-term TCM preconditioning.

Initially, the morphology of fibroblasts and MSCs was similar; however, after prolonged TCM treatment, MSCs acquired an elongated spindle-shape morphology, and 3T3 and primary fibroblasts maintained their original shape (Fig. 2C–F). This morphological change in MSCs coincided with the extension of linear actin filaments and microtubules (Fig. 3A). These changes further support the importance of RhoA, as activation of Rho effector mDia1 induces cell elongation coupled with parallel alignment of linear actin and microtubules [51].

The prolonged exposure of MSCs to TCM also resulted in visible loss of stress fibers (Fig. 3B) and focal adhesions (Fig. 4A–C). These changes correlate with increased expression of Cdc42, which can block Rho-induced focal adhesions and stress fibers [52]. Furthermore, Cdc42 is crucial in cell elongation processes, such as neurite outgrowth [53]. ANOVA revealed that only Cdc42 shows a significant interaction effect between cell type and treatment, suggesting it is a primary contributor to the long-term differences.

Two unique obstacles in MSC homing as compared to fibroblasts include extravasation from the bone marrow to enter circulation and intravasation into the site of inflammation [54]. The differential cellular elongation could aid as cells squeeze through the basement membrane. Since undifferentiated MSCs have a more deformable nucleus, they are better suited for this task [55]. Our results indicate that MSC nuclei elongate in response to TCM (Fig. 3D). In contrast to fibroblasts, MSCs have the ability to squeeze through 3 µm pore transwell inserts, which are approximately 3-fold smaller than the cell diameter (Fig. 7A). The up-regulation of Cdc42 may also prove beneficial in this process, as it is critical for nuclear translocation [56].

These results elucidate a fundamental difference in the recruitment of fibroblasts and MSCs to tumors by utilizing a unique set of mechanical changes to overcome their extensive physiological barriers. Furthermore, this new knowledge may be used in the development of novel therapeutics. Understanding the mechanisms of MSC homing can be used for more successful tumor targeting. Further studies can be focused on discovering the complex interactions of Rho GTPases and their effectors which modulate these cell-specific mechanical changes.

## Materials and Methods

### Materials

Cellgro IMDM, RPMI 1640, DMEM, L-glutamine, sodium bicarbonate, PBS, and penicillin-streptomycin were purchased from Mediatech. FBS was purchased from Atlanta Biologicals and Type I, II collagenase from Worthington Biochemicals. Rat anti-mouse PE-Sca1, PerCP-CD45, PE-CD29, and APC-CD11b, and red blood cell lysis buffer were purchased from Biolegend. Rhodamine-Phalloidin, rabbit vinculin monoclonal antibody, Alexa Fluor 488 anti-rabbit IgG, FITC-conjugated mouse anti-alpha tubulin, and Fluospheres carboxylate-modified 100 nm particles (F8801) were purchased from Invitrogen. Cy3-SMA was purchased from Sigma. PDS-1000 Biolistic Helium Particle Injection, 1800 psi rupture discs, and macrocarriers were purchased from BioRad. Primers for gene analysis were obtained from IDT. All other reagents were purchased from VWR or Sigma-Alrich unless otherwise specified.

### Cell Culture

Swiss 3T3 fibroblasts and 4T1 mammary carcinoma cells were purchased from American Type Cell Culture and cultured according to manufacturer's protocol. MSCs were isolated from murine bone marrow and cultured in MSC growth media (IMDM supplemented with 20% FBS, 2 mM L-glutamine, 100 U/ml penicillin, and 100 U/ml streptomycin). Bone marrow was extracted from the femurs and tibias of 8–10 week old Balb/C mice (Charles River Laboratories, Wilmington, MA) by crushing the bones in FBS solution (1 mg/ml Type I collagenase in 30% FBS and 70% PBS), filtering the cell suspension using a 70-µm cell strainer, and centrifuging at 2000× g for 10 minutes. The bone marrow cells were resuspended in MSC growth media and seeded at 3×10^6^ cells/cm^2^. After 24 hours, non-adherent cells were removed, and cells were cultured in MSC growth media (replaced 3 times per week). Differentiation was induced with either adipogenic or osteogenic media per standard protocols [33]. See Information S1 for details of kidney fibroblast isolation. All animal studies were approved by the Institutional Animal Care and Use Committee at Georgia Institute of Technology.

### Flow Cytometry

To determine cell phenotype, cells were analyzed with a LSR-II flow cytometer. Briefly, cells were detached, centrifuged, and separated into 100 µl aliquots then labeled with either 2% PerCP-CD45, PE-Sca1, and APC-CD11b antibodies or PE-CD61, FITC-CD29, and APC-VCAM1. A negative control for each cell type was used to determine positive populations. All studies were performed in triplicate with n = 100,000 events per sample.

### Conditioned Media Collection

Murine mammary carcinoma 4T1 cells were cultured to confluency in 10 cm dishes with standard growth media, then washed in PBS, and incubated in 8 mL unsupplemented DMEM for 24 hours. Cell-free TCM was obtained by centrifugation and filtration. TCM was prepared in a single batch, aliquoted and frozen at −80°C for future use. For all assays with TCM, control media was unsupplemented DMEM.

### 3D Co-Culture Model

To create the 3D ‘sandwich’ model either 4T1 or MSCs were first plated in a 24-well plate at a density of 10,000 cells/cm^2^. Next, 500 µl of a 2 mg/ml collagen solution (pH 7.4) was added to the top of the first cell layer. The collagen solution was prepared by diluting 4.0 mg/ml type I rat tail collagen (BD Bioscience) with serum-free DMEM and buffering solutions. After the collagen was polymerized, fluorescently-labeled MSCs (stained with CM-DiI (Invitrogen) as previously described [57]) were added to the top of the collagen gel (10,000 cells/cm^2^), and the plate was incubated overnight before imaging with the Nikon Eclipse Ti inverted epifluorescent microscope (Fig. 2G,H).

### Microrheological Characterization

Multiple particle tracking microrheology (MPT) was used to measure the mechanical properties within the cytoplasm of living cells [58]. With this technique, the thermal displacements of fluorescent probe particles are monitored with a fluorescent microscope and related to the viscoelastic properties of the fluid surrounding the tracked particles using the Stokes-Einstein equation [26].

For these studies, cells were cultured to 60–80% confluency on 35 mm glass bottom dishes. Fluorescent 100 nm microspheres, to be used as mechanical probes, were diluted 1∶10 in 100% ethanol and dialyzed overnight against 100% ethanol at 4°C. The microsphere solutions were then equilibrated to room temperature, sonicated and briefly centrifuged, to remove particle aggregates. 20 µL of the particle solution was then added to the surface of each macrocarrier. The macrocarriers were dried overnight in the dark and used the next day to inject microspheres into the cell cytoplasm with the PDS-1000 Biolistic Helium Particle Injection System and 1800 psi rupture discs. After injection, microspheres on the cell surface were removed by extensive washing with PBS, and the cells were incubated overnight in growth media. The following day growth media was replaced with serum-free media, which was used in all experiments.

Injected cells were placed on the stage of a Nikon Eclipse Ti inverted epifluorescent microscope, which was maintained at 37°C and 5% carbon dioxide throughout the experiment using an In Vivo Scientific environmental cell chamber. Twenty-second videos of particle diffusion in cells were collected at 33 ms temporal resolution (30 frames per second) with the Photometrics QuantEM CCD camera (Princeton Instruments). The Nikon CFI Apochromat TIRF 100× oil-immersion lens was used for particle tracking. With this lens, which has a high numerical aperature (NA = 1.49) and a reduced signal to noise ratio, it was possible to obtain 5 nm spatial resolution. This value was determined by tracking particles immobilized on a glass coverslip with a strong adhesive. High spatial resolution is obtained by tracking the 2D location of the intensity-weighted centroid of a particle that remains in the focal plane over the 20-second period. A custom MPT routine incorporated in the MetaMorph software (Molecular Devices; Downington, PA) was used to simultaneously monitor the coordinates of 10–20 particles per video. For each condition, particles were tracked in a minimum of 12 cells using 2–3 plates per condition.

Particle tracking data was analyzed using a custom routine written for MATLAB software. Briefly, the coordinates of the particle centroids were transformed into families of time-averaged mean squared displacements (MSDs), with MSD defined as <Δr^2^(Δt)> = <[x(t+Δt)−x(t)]^2^+[y(t+Δt)−y(t)]^2^>. The ensemble-averaged time-dependent MSDs (<<Δr^2^(Δt)>>), reported in Figure 5A–B, were then calculated from the individual particle time-averaged MSDs (<Δr^2^(Δt)>). The mechanical properties of the viscoelastic cytoplasm were then determined from the amplitude, <<Δr^2^(ω)>>, and logarithmic slope, α(ω) = d ln <<Δr^2^(t)>>/d ln t, of the ensemble-averaged time-dependent MSDs ((<Δr^2^(Δt)>)), where ω = 1/t [59]. To facilitate this analysis, the Stokes-Einstein equation was written in the Laplace domain, where the viscoelastic modulus was defined as G(s) = k_B_T/πas<<Δr^2^(s)>> (k_B_ = Boltzman constant, T = absolute temperature, s = Laplace frequency, a = particle radius). The frequency-dependent complex shear modulus (G^*^(ω)) was then determined from the projection of G(s) in the Fourier domain. The real and imaginary components of the complex shear modulus (G^*^(ω)), which are the viscous (G″(ω)) and elastic (G′(ω)) moduli, respectively, were reported in Figure 5. The phase angle (δ, where δ = arctan (G″(ω))/G′(ω)) was used to characterize the degree of stiffness (Fig. 5D), where δ = 90° for a viscous liquid, δ = 0° for a Hookean solid, 0°<δ<90° for a viscoelastic material.

### Centrifugal Force Adhesion Assay

Cells were seeded at 10,000 cells/cm^2^ in normal growth media and cultured to 80% confluency in a tissue culture treated 96-well plate. Normal growth media was removed and cells were then incubated in serum-free TCM or CM for 24 hours. Cells were then treated with 2 µM Calcein AM (Anaspec), a transmembrane fluorescent viability marker, in PBS+2 mM dextrose for 20 minutes at 37°C. Cells were then carefully rinsed and covered with PBS-dextrose before an initial emission reading was taken at 485 nm excitation, 535 nm emission on a DTX-800 Multimode Detector microwell plate reader. (Beckman Coulter). Next, sealing tape (Nalge Nunc) was applied to the plate before inverting it and centrifuging it at 600 RPM (64 g) for 5 minutes in a Beckman Coulter Allegra 25R centrifuge (TS-5.1-500 rotor). Wells were then carefully aspirated and rinsed to remove any floating cells, again PBS-dextrose was added and a final emission reading was taken. Adherent fraction was calculated by normalizing the final florescence with the pre-spin values [60].

### Immunoflourescence Assays

For visualization of cytoskeletal proteins, cells cultured on glass cover slips were briefly extracted in a buffer containing 80 mM PIPES (pH 6.8), 1 mM MgCl_2_, 5 mM EDTA, and 0.5% Triton X-100 before fixation with 0.5% glutaraldehyde in PBS. The reaction was quenched with 1 mg/mL sodium borohydride, before permeabilization with Triton X-100 and blocking with FBS. Cells were stained for one hour at room temperature with either 1∶50 FITC-conjugated anti-tubulin, 1∶200 Rhodamine Phalloidin , or 1 µg/mL rabbit anti-vinculin followed by 1∶1000 goat anti-rabbit Alexa Flour 488 before sealing with Vectashield (Vector Labs) containing DAPI. For morphological visualization, cells were stained with crystal violet then rinsed extensively. All cells were visualized using either an inverted Nikon Eclipse Ti or Zeiss LSM 510 UV confocal microscope.

### Quantitative Image Analysis

Quantitative image analysis was performed by a series of custom written MATLAB algorithms. For cell elongation, borders were manually drawn around cells before segmentation to extract cell major and minor axis. After normalization for background, stress fibers were segmented by a Laplacian filter, and then normalized to cell area extracted from a built-in MATLAB thresholding algorithm. Nuclei were analyzed using a semi-automated MATLAB algorithm that visualized each image before quantification to ensure only single nuclei were measured. Similarly, for actin and vinculin quantification, images were normalized then segmented for quantification.

### Migration Assays

To quantify migration, 3 or 8 µm pore transwell inserts (Greiner Bio One) were added to a 24 well plate, rinsed in PBS then incubated overnight in serum free DMEM. The following day, the media in the well was replaced with 600 µL of CM or TCM and 10,000 serum-starved MSCs in 100 µL of CM or TCM were added to the top of each insert. After desired incubation time, non-migrated cells were gently removed from the top of each transwell using a cotton swab. The cells on the bottom of the inserts were then fixed in 4% formaldehyde with 0.01%Triton X-100 and stained with DAPI. For quantification, an automated image acquisition algorithm collected 9 fields of view at 10× magnification per transwell and nuclei were counted using standard functions in MetaMorph software. The average number of cells per image was reported.

### Gene Expression Analysis

Total RNA was isolated using TRIzol reagent (Invitrogen) per manufacturer's protocol before reverse transcription using the iScript cDNA synthesis kit (BioRad). DNA concentration and quality were verified by spectroscopy. To verify differentiation, PCR was run per previous literature [34]. For qRT-PCR on the small Rho GTPases, Cdc42, RhoA, and Rac1, as well as an endogenous GAPDH control, the real-time PCR reaction was run using SsoAdvanced SYBR Green Supermix (BioRad) in an AB Step One Plus thermocycler (n = 5). Primer sequences are reported in Information S2. Values are reported as fold change in expression over fibroblasts in CM ± S.E.M. after normalization to respective endogenous GAPDH control.

### Statistics

All studies were performed in triplicate (unless otherwise indicated). Statistical analysis was carried out using a student's t-test for comparison between two groups or analysis of variance (ANOVA) to compare effects between cell types, considering p<0.05 to be significant (***p<0.001,**p<0.01,*p<0.05). Data were reported as the mean ± s.e.m.

## Supporting Information

Table S1 **Acronyms of Soluble Factors.**
(DOC)

Information S1 **Kidney Fibroblast Isolation and Characterization.**
(DOC)

Information S2 **Tables of PCR Primers.**
(DOC)
