# Supplementary material for: Differential Mechanical Response of Mesenchymal Stem Cells and Fibroblasts to Tumor-Secreted Soluble Factors
Source: PLoS One. 2012 Mar 16;7(3):e33248. doi: 10.1371/journal.pone.0033248 (PMC3306382; doi:10.1371/journal.pone.0033248)
Supplement: Table S1 — Acronyms of Soluble Factors. (DOC) [file pone.0033248.s001.doc]

**Table S1.** Acronyms of Soluble Factors

|  | Full Name | Abbreviation |
| --- | --- | --- |
| Growth Factors | Angiopoietin 2 | Ang-2 |
|  | Basic fibroblast growth factor | bFGF/FGF-2 |
|  | Hepatocyte growth factor | HGF |
|  | Platelet-derived growth factor | PDGF |
|  | Transforming growth factor β1 | TGF-β1 |
|  | Vascular endothelial growth factor | VEGF |
|  | Monocyte chemotactic protein 1 | CCL2/MCP-1 |
| Chemokines  &  Cytokines | Regulated upon Activation, Normal T-cell Expressed, and Secreted | CCL5/RANTES |
|  | Monocyte-specific chemokine 3 | CCL7/MCP3 |
|  | Melanoma growth stimulating activity α | CXCL1/Gro-α |
|  | Growth-regulated protein β | CXCL2/Gro-β |
|  | Stromal cell-derived factor 1 | CXCL12/SDF1 |
|  | n/a | CXCL16 |
|  | Colony stimulating factor | CSF |
|  | Interleukin | IL |
|  | Tumor necrosis factor-α | TNF-α |
