# Supplementary material for: Differential Mechanical Response of Mesenchymal Stem Cells and Fibroblasts to Tumor-Secreted Soluble Factors
Source: PLoS One. 2012 Mar 16;7(3):e33248. doi: 10.1371/journal.pone.0033248 (PMC3306382; doi:10.1371/journal.pone.0033248)

**Supporting Information S1**

**Isolation and Characterization of Kidney Fibroblasts.** Primary fibroblasts were isolated from the kidneys of 8-10 week old Balb/C mice. For these studies, isolated kidneys were minced and then digested at 37°C for 45 minutes with a cocktail of 2 mg/ml type I and 2 mg/ml type II collagenase. After digestion, cells were filtered through a 70-µm cell strainer, washed in PBS, and centrifuged at 800 x g. Within 2 passages, cells exhibited a fibroblast-like morphology. To confirm phenotype, cells were stained for fibroblast markers α-smooth muscle actin and CD29. For immunostaining, cells were fixed in 4% formaldehyde, permeabilized with 0.5% Triton-X100, then blocked with 5% normal horse serum before incubation with 1:200 Cy3 α-SMA and 1:500 FITC CD29 (Fig. 1D). Primary fibroblasts were cultured for up to 6 passages in RPMI 1640 supplemented with 20% fetal bovine serum, 1% L-glutamine, 100 U/ml penicillin, and 100 U/ml streptomycin.


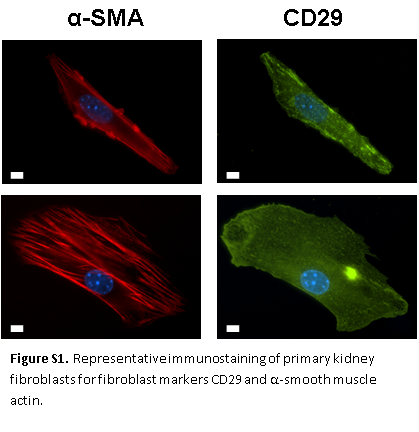

Supplement: Information S1 — Kidney Fibroblast Isolation and Characterization. (DOC) [file pone.0033248.s002.doc]
