# Supplementary material for: Differential Mechanical Response of Mesenchymal Stem Cells and Fibroblasts to Tumor-Secreted Soluble Factors
Source: PLoS One. 2012 Mar 16;7(3):e33248. doi: 10.1371/journal.pone.0033248 (PMC3306382; doi:10.1371/journal.pone.0033248)
Supplement: Information S2 — Tables of PCR Primers. (DOC) [file pone.0033248.s003.doc]

**Supporting Information S2**

**Table S2.** Primers for qRT-PCR

| Gene | Primer Nucleotide Sequence | | Accession Number |  |
| --- | --- | --- | --- | --- |
| Cdc42 | Forward 5′  Reverse 5′ | CCCATCGGAATATGTACCAACTG  CCAAGAGTGTATGGCTCTCCAC | L78075 | |
| RhoA | Forward 5′  Reverse 5′ | AGCTTGTGGTAAGACATGCTTG  GTGTCCCATAAAGCCAACTCTAC | NM 016802 | |
| Rac1 | Forward 5′  Reverse 5′ | GAGACGGAGCTGTTGGTAAAA  ATAGGCCCAGATTCACTGGTT | BC003828 | |
| GAPDH | Forward 5′  Reverse 5′ | AGGTCGGTGTGAACGGATTTG  TGTAGACCATGTAGTTGAGGTCA | NM 008084 | |

**Table S3.** Primers for RT-PCR

| Gene | Primer Nucleotide Sequence | | Accession Number |  |
| --- | --- | --- | --- | --- |
| Adipsin | Forward 5′  Reverse 5′ | CTGCTGGACGAGCAGTGG GATGACACTCGGGTATAGACGC | NM 001928 | |
| Osteocalcin | Forward 5′  Reverse 5′ | TCT GCT CAC TCT GCT GAC GGA GCT GCT GTG ACA TCC | NM 007541 | |
| GAPDH | Forward 5′  Reverse 5′ | AGGTCGGTGTGAACGGATTTG  TGTAGACCATGTAGTTGAGGTCA | NM 008084 | |
